# Machine Learning in Schizophrenia: A Systematic Review and Meta-Analysis of Diagnostic and Predictive Models

**DOI:** 10.1192/bjo.2025.10148

**Published:** 2025-06-20

**Authors:** Oluwatobi Idowu, Nicholas Aderinto, Gbolahan Olatunji, Emmanuel Kokori

**Affiliations:** 1 Cheshire and Wirral Partnership NHS Foundation Trust, Chester, United Kingdom; 2 Department of Medicine, Ladoke Akintola University of Technology, Ogbomoso, Nigeria; 3 Department of Medicine and Surgery, University of Ilorin, Ilorin, Nigeria

## Abstract

**Aims:** Schizophrenia is a psychiatric disorder characterized by diverse clinical presentations, posing challenges in early diagnosis and prognosis. Machine learning (ML) has emerged as a promising tool to enhance diagnostic accuracy, predict disease progression, and personalize treatment strategies. This systematic review and meta-analysis synthesized current evidence on the application of ML in schizophrenia diagnosis, prognosis, and treatment response prediction.

**Methods:** A search was conducted across databases including PubMed, Embase, Scopus, Web of Science, and IEEE Xplore, adhering to PRISMA guidelines. Studies employing ML algorithms for schizophrenia classification, risk prediction, or treatment response modelling were included. Extracted data encompassed ML model types, sample sizes, data modalities (e.g., neuroimaging, clinical, genetic), and performance metrics such as accuracy, sensitivity, specificity, and area under the curve (AUC). A meta-analysis was performed to estimate pooled diagnostic performance, with heterogeneity assessed using I² statistics and publication bias evaluated via funnel plots and Egger’s test.

**Results:** A total of 31 studies involving task-based functional MRI (t-fMRI) data were included in the meta-analysis. The pooled sensitivity and specificity for ML-based schizophrenia classification were both 0.83 (95% CI: 0.78–0.88), indicating a high level of diagnostic accuracy. Notably, studies focusing on selective attention tasks demonstrated higher specificity (0.86) compared with those assessing working memory tasks (0.79). Significant heterogeneity (I² = 72%) was observed, attributable to variations in neuropsychological domains, participant demographics, and clinical features.

**Conclusion:** Machine learning exhibits substantial potential in improving schizophrenia diagnosis and outcome prediction, particularly when utilizing task-based neuroimaging data. However, challenges related to data heterogeneity, external validation, and clinical implementation persist. Future research should focus on standardizing ML methodologies, integrating multi-modal data, and enhancing model interpretability to facilitate translation into clinical psychiatry.

